# (*E*)-2-Bromo-4-[(1,5-dimethyl-3-oxo-2-phenyl-2,3-dihydro-1*H*-pyrazol-4-yl)imino­meth­yl]-6-meth­oxy­phenyl 4-methyl­benzene­sulfonate

**DOI:** 10.1107/S160053681201447X

**Published:** 2012-04-06

**Authors:** Zhong-Yu Duan, Lin Yang, Li-Ping Yang, Xiu-Wu Liu

**Affiliations:** a College of Chemical Engineering, Hebei University of Technology, Tianjin 300130, People’s Republic of China

## Abstract

In the title compound, C_26_H_24_BrN_3_O_5_S, the central benzene ring makes dihedral angles of 6.27 (6), 33.63 (6) and 69.31 (5)°, respectively, with the pyrazolone ring, the bromo­benzene ring and the terminal phenyl ring. An intra­molecular C—H⋯O hydrogen bond occurs. The crystal packing features weak non-classical C—Br⋯O inter­actions [Br⋯O = 3.222 (2) Å] that form inversion-related dimers.

## Related literature

For general background to the use of Schiff base derivatives in the development of protein and enzyme mimics, see: Santos *et al.* (2001 ▶). For closely related crystal structures, see: Chen & Yu (2006 ▶); Han *et al.* (2008 ▶). For reference bond-length data, see: Allen *et al.* (1987 ▶).
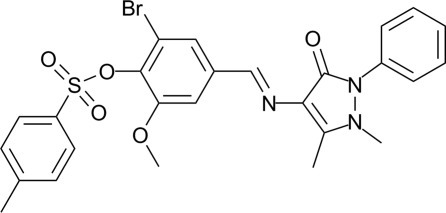


## Experimental

### 

#### Crystal data

C_26_H_24_BrN_3_O_5_S
*M* *_r_* = 570.45
Monoclinic, 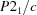

*a* = 10.210 (2) Å
*b* = 20.364 (5) Å
*c* = 12.171 (3) Å
β = 90.906 (4)°
*V* = 2530.2 (10) Å^3^
*Z* = 4
Mo *K*α radiation
μ = 1.75 mm^−1^
*T* = 294 K
0.25 × 0.20 × 0.13 mm

#### Data collection

Bruker SMART APEX CCD area-detector diffractometer
Absorption correction: multi-scan (*SADABS*; Sheldrick, 1996 ▶) *T*_min_ = 0.653, *T*_max_ = 0.797
21416 measured reflections
4462 independent reflections
3998 reflections with *I* > 2σ(*I*)
*R*_int_ = 0.039

#### Refinement

*R*[*F* ^2^ > 2σ(*F* ^2^)] = 0.029
*wR*(*F* ^2^) = 0.071
*S* = 1.02
4462 reflections
329 parameters
H-atom parameters constrained
Δρ_max_ = 0.35 e Å^−3^
Δρ_min_ = −0.36 e Å^−3^

### 

Data collection: *SMART* (Bruker, 1999 ▶); cell refinement: *SAINT* (Bruker, 1999 ▶); data reduction: *SAINT*; program(s) used to solve structure: *SHELXS97* (Sheldrick, 2008 ▶); program(s) used to refine structure: *SHELXL97* (Sheldrick, 2008 ▶); molecular graphics: *SHELXTL* (Sheldrick, 2008 ▶); software used to prepare material for publication: *SHELXTL*.

## Supplementary Material

Crystal structure: contains datablock(s) I, global. DOI: 10.1107/S160053681201447X/ds2184sup1.cif

Structure factors: contains datablock(s) I. DOI: 10.1107/S160053681201447X/ds2184Isup2.hkl

Supplementary material file. DOI: 10.1107/S160053681201447X/ds2184Isup3.cml

Additional supplementary materials:  crystallographic information; 3D view; checkCIF report

## Figures and Tables

**Table 1:** Hydrogen-bond geometry (Å, °)

*D*—H⋯*A*	*D*—H	H⋯*A*	*D*⋯*A*	*D*—H⋯*A*
C14—H14⋯O5	0.93	2.30	2.991 (2)	131

**Fig. 1.**
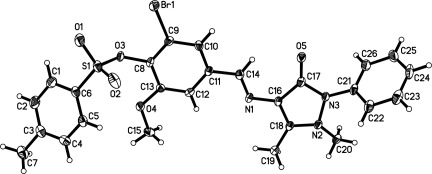
The structure of (I), with displacement ellipsoids for non-H atoms drawn at the 50% probability level.

**Fig. 2.**
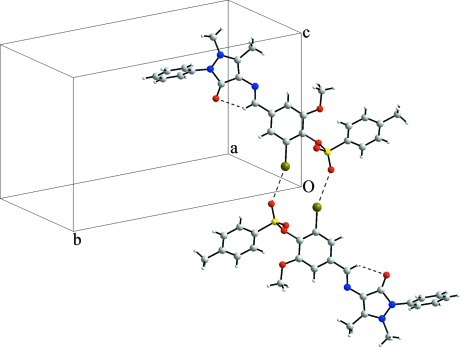
A packing diagram for (I), with hydrogen bonds drawn as dashed lines.

## supplementary crystallographic information

### Comment

There has been steady growth of interest in the synthesis, structure, and reactivity of Schiff bases due to their potentially biological activities such as protein and enzyme mimics (Santos *et al.*, 2001). Among the large number of compounds, 4-amino-1,5-dimethyl-2-phenylpyrazol-3-one forms a variety of Schiff bases with aldehydes, and the synthesis and crystal structures of some of them, such as (*E*)-5-(1,5-Dimethyl-3-oxo-2-phenyl-2,3-dihydro- 1*H*-pyrazol-4-yliminomethyl)-2-methoxyphenyl benzenesulfonate (Chen & Yu, 2006) and (*E*)-4-((1,5-Dimethyl-3-oxo-2-phenyl-2,3-dihydro-1*H*-pyrazol-4-ylimino) methyl)phenyl 4-bromobenzenesulfonate (Han *et al.*, 2008) have been reported.

Structural information is useful when investigating the coordination properties of Schiff bases functioning as ligands. We report here the synthesis and molecular structure of the title Schiff base compound, (I), (Fig. 1)

In the title molecule (Fig. 1), bond lengths are within normal ranges (Allen *et al.*, 1987). The pyrazolone ring (C16—C18/N1—N3/O5) is almost planar, with an r.m.s. deviation for fitted atoms of 0.0426 Å. It makes a dihedral angle of 63.05 (6)° with the attached phenyl ring (C21—C26). The central benzene ring (C8—C14/O3/O4) is nearly planar, with an r.m.s. deviation for fitted atoms of 0.0559 Å. This group makes dihedral angles of 6.27 (6)°, 33.63 (6)° and 69.31 (5)°, respectively, with the the pyrazolone ring (C16—C18/N1—N3/O5), the bromobenzene ring (C1—C6) and the terminal phenyl ring (C21—C26).

An intramolecular C14—H14···O5═C17 hydrogen bond is found in (I) (Table 1, Fig. 2), which helps to stabilize the conformation of the molecule. The crystal packing is stabilized by weak, non-classical intermolecular C9—Br1···O1═S1 interactions (the Br···O distance and the C—Br···O angle, 3.222 Å and 147.62 °) that form inversion related dimers.

### Experimental

An anhydrous ethanol solution (50 ml) of 2-bromo-4-formyl-6-methoxyphenyl 4-methylbenzenesulfonate (3.85 g, 10 mmol) was added to an anhydrous ethanol solution (50 ml) of 4-amino-1,5-dimethyl-2-phenylpyrazol-3-one (2.03 g, 10 mmol) and the mixture stirred at 350 K for 3 h under N_2_, giving a yellow precipitate. The product was isolated, recrystallized from acetonitrile, and then dried in a vacuum to give pure compound (I) in 85% yield. Yellow single crystals of (I) suitable for X-ray analysis were obtained by slow evaporation of an acetonitrile solution.

### Refinement

The H atoms were included in calculated positions and refined using a riding model approximation. Constrained C—H bond lengths and isotropic U parameters: 0.93 Å and *U*_iso_(H) = 1.2*U*_eq_(C) for C*sp*^2^—H; 0.96 Å and *U*_iso_(H) = 1.5*U*_eq_(C) for methyl C—H.

### Crystal data

**Table:**

C_26_H_24_BrN_3_O_5_S	*F*(000) = 1168
*M**_r_* = 570.45	*D*_x_ = 1.497 Mg m^−^^3^
Monoclinic, *P*2_1_/*c*	Mo *K*α radiation, λ = 0.71073 Å
Hall symbol: -P 2ybc	Cell parameters from 8917 reflections
*a* = 10.210 (2) Å	θ = 1.9–27.9°
*b* = 20.364 (5) Å	µ = 1.75 mm^−^^1^
*c* = 12.171 (3) Å	*T* = 294 K
β = 90.906 (4)°	Block, yellow
*V* = 2530.2 (10) Å^3^	0.25 × 0.20 × 0.13 mm
*Z* = 4	

### Data collection

**Table:**

Bruker SMART APEX CCD area-detector diffractometer	4462 independent reflections
Radiation source: fine-focus sealed tube	3998 reflections with *I* > 2σ(*I*)
Graphite monochromator	*R*_int_ = 0.039
φ and ω scans	θ_max_ = 25.0°, θ_min_ = 2.0°
Absorption correction: multi-scan (*SADABS*; Sheldrick, 1996)	*h* = −12→10
*T*_min_ = 0.653, *T*_max_ = 0.797	*k* = −24→24
21416 measured reflections	*l* = −14→14

### Refinement

**Table:**

Refinement on *F*^2^	Primary atom site location: structure-invariant direct methods
Least-squares matrix: full	Secondary atom site location: difference Fourier map
*R*[*F*^2^ > 2σ(*F*^2^)] = 0.029	Hydrogen site location: inferred from neighbouring sites
*wR*(*F*^2^) = 0.071	H-atom parameters constrained
*S* = 1.02	*w* = 1/[σ^2^(*F*_o_^2^) + (0.0428*P*)^2^] where *P* = (*F*_o_^2^ + 2*F*_c_^2^)/3
4462 reflections	(Δ/σ)_max_ < 0.001
329 parameters	Δρ_max_ = 0.35 e Å^−^^3^
0 restraints	Δρ_min_ = −0.36 e Å^−^^3^

### Special details

**Table:**

Geometry. All e.s.d.'s (except the e.s.d. in the dihedral angle between two l.s. planes) are estimated using the full covariance matrix. The cell e.s.d.'s are taken into account individually in the estimation of e.s.d.'s in distances, angles and torsion angles; correlations between e.s.d.'s in cell parameters are only used when they are defined by crystal symmetry. An approximate (isotropic) treatment of cell e.s.d.'s is used for estimating e.s.d.'s involving l.s. planes.
Refinement. Refinement of *F*^2^ against ALL reflections. The weighted *R*-factor *wR* and goodness of fit *S* are based on *F*^2^, conventional *R*-factors *R* are based on *F*, with *F* set to zero for negative *F*^2^. The threshold expression of *F*^2^ > 2σ(*F*^2^) is used only for calculating *R*-factors(gt) *etc*. and is not relevant to the choice of reflections for refinement. *R*-factors based on *F*^2^ are statistically about twice as large as those based on *F*, and *R*- factors based on ALL data will be even larger.

### Fractional atomic coordinates and isotropic or equivalent isotropic displacement parameters (Å^2^)

**Table:**

	*x*	*y*	*z*	*U*_iso_*/*U*_eq_	
Br1	0.19479 (2)	0.007075 (10)	0.092923 (16)	0.03095 (9)	
S1	−0.07113 (5)	−0.09236 (2)	0.21545 (4)	0.02290 (13)	
N1	0.42286 (15)	0.06686 (7)	0.58171 (12)	0.0162 (3)	
N2	0.58362 (15)	0.16776 (7)	0.78091 (12)	0.0189 (4)	
N3	0.63838 (16)	0.19415 (7)	0.68517 (12)	0.0196 (4)	
O1	−0.08922 (15)	−0.10227 (8)	0.10047 (11)	0.0384 (4)	
O2	−0.10940 (13)	−0.03239 (7)	0.26545 (12)	0.0316 (4)	
O3	0.08380 (12)	−0.10214 (6)	0.23741 (10)	0.0187 (3)	
O4	0.09160 (13)	−0.11147 (6)	0.45433 (10)	0.0223 (3)	
O5	0.60059 (14)	0.18166 (6)	0.49768 (10)	0.0251 (3)	
C1	−0.1563 (2)	−0.21849 (10)	0.23038 (16)	0.0311 (5)	
H1	−0.1283	−0.2236	0.1586	0.037*	
C2	−0.2158 (2)	−0.26989 (10)	0.28479 (17)	0.0335 (5)	
H2	−0.2259	−0.3101	0.2494	0.040*	
C3	−0.26054 (19)	−0.26255 (10)	0.39108 (16)	0.0241 (5)	
C4	−0.24054 (19)	−0.20296 (10)	0.44337 (16)	0.0235 (4)	
H4	−0.2684	−0.1976	0.5152	0.028*	
C5	−0.17980 (18)	−0.15113 (9)	0.39069 (16)	0.0220 (4)	
H5	−0.1667	−0.1113	0.4267	0.026*	
C6	−0.13930 (18)	−0.15935 (9)	0.28468 (15)	0.0201 (4)	
C7	−0.3296 (2)	−0.31719 (10)	0.45012 (18)	0.0317 (5)	
H7A	−0.2661	−0.3448	0.4863	0.048*	
H7B	−0.3795	−0.3427	0.3981	0.048*	
H7C	−0.3872	−0.2989	0.5036	0.048*	
C8	0.15558 (18)	−0.05559 (9)	0.29815 (14)	0.0163 (4)	
C9	0.22147 (19)	−0.00618 (9)	0.24505 (16)	0.0191 (4)	
C10	0.30593 (18)	0.03464 (9)	0.30430 (15)	0.0181 (4)	
H10	0.3524	0.0673	0.2685	0.022*	
C11	0.32058 (18)	0.02652 (9)	0.41681 (14)	0.0165 (4)	
C12	0.24861 (18)	−0.02151 (9)	0.47152 (15)	0.0184 (4)	
H12	0.2561	−0.0257	0.5474	0.022*	
C13	0.16597 (18)	−0.06287 (9)	0.41222 (14)	0.0164 (4)	
C14	0.41265 (18)	0.06955 (9)	0.47673 (15)	0.0183 (4)	
H14	0.4640	0.0990	0.4380	0.022*	
C15	0.0978 (2)	−0.12144 (11)	0.57034 (16)	0.0302 (5)	
H15A	0.1852	−0.1338	0.5918	0.045*	
H15B	0.0380	−0.1557	0.5901	0.045*	
H15C	0.0743	−0.0815	0.6071	0.045*	
C16	0.50319 (18)	0.11137 (9)	0.63731 (15)	0.0163 (4)	
C17	0.58248 (19)	0.16334 (9)	0.59291 (15)	0.0191 (4)	
C18	0.51022 (18)	0.11514 (9)	0.74984 (14)	0.0166 (4)	
C19	0.4502 (2)	0.07000 (9)	0.83058 (15)	0.0231 (4)	
H19A	0.5178	0.0461	0.8689	0.035*	
H19B	0.3932	0.0397	0.7927	0.035*	
H19C	0.4006	0.0950	0.8823	0.035*	
C20	0.65966 (19)	0.17428 (9)	0.88324 (14)	0.0213 (4)	
H20A	0.6175	0.1501	0.9405	0.032*	
H20B	0.6651	0.2198	0.9034	0.032*	
H20C	0.7462	0.1572	0.8729	0.032*	
C21	0.6892 (2)	0.25949 (9)	0.68276 (14)	0.0208 (4)	
C22	0.6206 (2)	0.31109 (9)	0.72901 (15)	0.0262 (5)	
H22	0.5433	0.3034	0.7664	0.031*	
C23	0.6693 (2)	0.37415 (10)	0.71839 (17)	0.0349 (6)	
H23	0.6244	0.4091	0.7493	0.042*	
C24	0.7828 (3)	0.38575 (10)	0.66286 (17)	0.0366 (6)	
H24	0.8134	0.4285	0.6551	0.044*	
C25	0.8518 (2)	0.33407 (11)	0.61842 (17)	0.0343 (6)	
H25	0.9294	0.3419	0.5816	0.041*	
C26	0.8049 (2)	0.27019 (10)	0.62896 (16)	0.0272 (5)	
H26	0.8513	0.2351	0.5999	0.033*	

### Atomic displacement parameters (Å^2^)

**Table:**

	*U*^11^	*U*^22^	*U*^33^	*U*^12^	*U*^13^	*U*^23^
Br1	0.04143 (16)	0.03531 (15)	0.01582 (12)	−0.01554 (10)	−0.00859 (9)	0.00683 (8)
S1	0.0207 (3)	0.0236 (3)	0.0241 (3)	−0.0062 (2)	−0.0083 (2)	0.0086 (2)
N1	0.0166 (9)	0.0142 (8)	0.0177 (8)	0.0016 (6)	−0.0030 (6)	−0.0042 (6)
N2	0.0256 (9)	0.0164 (8)	0.0145 (8)	−0.0026 (7)	−0.0030 (7)	−0.0006 (6)
N3	0.0268 (10)	0.0168 (9)	0.0149 (8)	−0.0060 (7)	−0.0029 (7)	−0.0001 (6)
O1	0.0418 (10)	0.0504 (10)	0.0225 (8)	−0.0242 (8)	−0.0163 (7)	0.0149 (7)
O2	0.0237 (8)	0.0179 (8)	0.0530 (10)	0.0017 (6)	−0.0022 (7)	0.0094 (7)
O3	0.0180 (7)	0.0191 (7)	0.0187 (7)	−0.0038 (6)	−0.0035 (5)	−0.0043 (5)
O4	0.0255 (8)	0.0257 (8)	0.0158 (7)	−0.0082 (6)	−0.0026 (6)	0.0015 (5)
O5	0.0362 (9)	0.0230 (8)	0.0159 (7)	−0.0093 (6)	−0.0046 (6)	0.0012 (5)
C1	0.0416 (14)	0.0297 (12)	0.0219 (11)	−0.0106 (10)	−0.0008 (9)	−0.0004 (9)
C2	0.0466 (15)	0.0223 (12)	0.0314 (13)	−0.0115 (10)	−0.0026 (10)	−0.0028 (9)
C3	0.0176 (11)	0.0222 (11)	0.0322 (12)	0.0007 (9)	−0.0053 (9)	0.0089 (8)
C4	0.0204 (11)	0.0242 (11)	0.0261 (11)	0.0023 (9)	0.0034 (8)	0.0054 (8)
C5	0.0195 (11)	0.0162 (11)	0.0301 (11)	0.0005 (8)	−0.0016 (9)	−0.0010 (8)
C6	0.0171 (11)	0.0203 (11)	0.0228 (10)	−0.0027 (8)	−0.0054 (8)	0.0057 (8)
C7	0.0298 (13)	0.0256 (12)	0.0395 (13)	−0.0042 (10)	−0.0038 (10)	0.0120 (9)
C8	0.0155 (10)	0.0157 (10)	0.0175 (9)	0.0010 (8)	−0.0043 (7)	−0.0044 (7)
C9	0.0217 (11)	0.0202 (10)	0.0152 (10)	0.0004 (8)	−0.0028 (8)	−0.0001 (7)
C10	0.0195 (11)	0.0144 (10)	0.0202 (10)	−0.0016 (8)	−0.0017 (8)	0.0012 (7)
C11	0.0167 (10)	0.0144 (10)	0.0184 (10)	0.0021 (8)	−0.0014 (8)	−0.0035 (7)
C12	0.0203 (11)	0.0210 (10)	0.0138 (10)	0.0022 (8)	−0.0004 (8)	−0.0022 (7)
C13	0.0158 (10)	0.0171 (10)	0.0163 (10)	0.0000 (8)	0.0001 (7)	0.0013 (7)
C14	0.0195 (11)	0.0138 (10)	0.0216 (10)	0.0015 (8)	0.0001 (8)	−0.0017 (7)
C15	0.0368 (13)	0.0337 (12)	0.0203 (11)	−0.0113 (10)	0.0039 (9)	0.0076 (9)
C16	0.0171 (10)	0.0128 (9)	0.0189 (10)	0.0007 (8)	−0.0022 (8)	−0.0018 (7)
C17	0.0219 (11)	0.0164 (10)	0.0187 (10)	0.0002 (8)	−0.0049 (8)	−0.0012 (7)
C18	0.0170 (10)	0.0142 (10)	0.0186 (10)	0.0025 (8)	−0.0015 (7)	−0.0030 (7)
C19	0.0256 (12)	0.0225 (11)	0.0214 (10)	−0.0031 (9)	0.0030 (8)	−0.0037 (8)
C20	0.0254 (11)	0.0235 (11)	0.0149 (10)	0.0001 (9)	−0.0048 (8)	−0.0030 (8)
C21	0.0314 (12)	0.0153 (10)	0.0153 (10)	−0.0036 (8)	−0.0102 (8)	0.0011 (7)
C22	0.0348 (13)	0.0202 (11)	0.0231 (11)	0.0002 (9)	−0.0104 (9)	0.0002 (8)
C23	0.0557 (16)	0.0216 (12)	0.0270 (12)	0.0010 (11)	−0.0142 (11)	−0.0011 (9)
C24	0.0644 (18)	0.0197 (12)	0.0251 (12)	−0.0170 (11)	−0.0199 (12)	0.0042 (9)
C25	0.0426 (14)	0.0348 (13)	0.0253 (12)	−0.0190 (11)	−0.0063 (10)	0.0033 (9)
C26	0.0346 (13)	0.0234 (11)	0.0235 (11)	−0.0056 (9)	−0.0036 (9)	−0.0003 (8)

### Geometric parameters (Å, º)

**Table:**

Br1—C9	1.8866 (19)	C9—C10	1.391 (3)
S1—O2	1.4220 (15)	C10—C11	1.385 (2)
S1—O1	1.4230 (15)	C10—H10	0.9300
S1—O3	1.6125 (13)	C11—C12	1.398 (3)
S1—C6	1.7533 (19)	C11—C14	1.470 (3)
N1—C14	1.281 (2)	C12—C13	1.387 (2)
N1—C16	1.391 (2)	C12—H12	0.9300
N2—C18	1.358 (2)	C14—H14	0.9300
N2—N3	1.407 (2)	C15—H15A	0.9600
N2—C20	1.463 (2)	C15—H15B	0.9600
N3—C17	1.400 (2)	C15—H15C	0.9600
N3—C21	1.429 (2)	C16—C18	1.373 (2)
O3—C8	1.402 (2)	C16—C17	1.443 (3)
O4—C13	1.353 (2)	C18—C19	1.485 (3)
O4—C15	1.427 (2)	C19—H19A	0.9600
O5—C17	1.234 (2)	C19—H19B	0.9600
C1—C6	1.383 (3)	C19—H19C	0.9600
C1—C2	1.384 (3)	C20—H20A	0.9600
C1—H1	0.9300	C20—H20B	0.9600
C2—C3	1.387 (3)	C20—H20C	0.9600
C2—H2	0.9300	C21—C26	1.377 (3)
C3—C4	1.384 (3)	C21—C22	1.387 (3)
C3—C7	1.506 (3)	C22—C23	1.384 (3)
C4—C5	1.387 (3)	C22—H22	0.9300
C4—H4	0.9300	C23—C24	1.372 (3)
C5—C6	1.371 (3)	C23—H23	0.9300
C5—H5	0.9300	C24—C25	1.381 (3)
C7—H7A	0.9600	C24—H24	0.9300
C7—H7B	0.9600	C25—C26	1.393 (3)
C7—H7C	0.9600	C25—H25	0.9300
C8—C9	1.377 (3)	C26—H26	0.9300
C8—C13	1.399 (2)		
			
O2—S1—O1	120.69 (9)	C11—C12—H12	120.1
O2—S1—O3	108.10 (7)	O4—C13—C12	125.98 (16)
O1—S1—O3	104.91 (8)	O4—C13—C8	114.79 (15)
O2—S1—C6	110.41 (9)	C12—C13—C8	119.23 (17)
O1—S1—C6	108.42 (9)	N1—C14—C11	120.69 (17)
O3—S1—C6	102.71 (8)	N1—C14—H14	119.7
C14—N1—C16	119.69 (16)	C11—C14—H14	119.7
C18—N2—N3	107.19 (14)	O4—C15—H15A	109.5
C18—N2—C20	126.28 (15)	O4—C15—H15B	109.5
N3—N2—C20	117.24 (15)	H15A—C15—H15B	109.5
C17—N3—N2	109.32 (15)	O4—C15—H15C	109.5
C17—N3—C21	122.99 (15)	H15A—C15—H15C	109.5
N2—N3—C21	121.46 (15)	H15B—C15—H15C	109.5
C8—O3—S1	120.47 (11)	C18—C16—N1	122.89 (17)
C13—O4—C15	117.56 (14)	C18—C16—C17	108.18 (15)
C6—C1—C2	118.85 (19)	N1—C16—C17	128.75 (16)
C6—C1—H1	120.6	O5—C17—N3	123.54 (17)
C2—C1—H1	120.6	O5—C17—C16	131.82 (16)
C1—C2—C3	121.21 (19)	N3—C17—C16	104.61 (15)
C1—C2—H2	119.4	N2—C18—C16	110.00 (16)
C3—C2—H2	119.4	N2—C18—C19	122.41 (16)
C4—C3—C2	118.37 (18)	C16—C18—C19	127.59 (16)
C4—C3—C7	119.69 (19)	C18—C19—H19A	109.5
C2—C3—C7	121.94 (18)	C18—C19—H19B	109.5
C3—C4—C5	121.24 (19)	H19A—C19—H19B	109.5
C3—C4—H4	119.4	C18—C19—H19C	109.5
C5—C4—H4	119.4	H19A—C19—H19C	109.5
C6—C5—C4	119.06 (18)	H19B—C19—H19C	109.5
C6—C5—H5	120.5	N2—C20—H20A	109.5
C4—C5—H5	120.5	N2—C20—H20B	109.5
C5—C6—C1	121.23 (18)	H20A—C20—H20B	109.5
C5—C6—S1	119.01 (15)	N2—C20—H20C	109.5
C1—C6—S1	119.70 (15)	H20A—C20—H20C	109.5
C3—C7—H7A	109.5	H20B—C20—H20C	109.5
C3—C7—H7B	109.5	C26—C21—C22	121.04 (19)
H7A—C7—H7B	109.5	C26—C21—N3	118.16 (18)
C3—C7—H7C	109.5	C22—C21—N3	120.72 (19)
H7A—C7—H7C	109.5	C23—C22—C21	118.7 (2)
H7B—C7—H7C	109.5	C23—C22—H22	120.6
C9—C8—C13	120.89 (16)	C21—C22—H22	120.6
C9—C8—O3	120.12 (16)	C24—C23—C22	120.9 (2)
C13—C8—O3	118.86 (16)	C24—C23—H23	119.5
C8—C9—C10	119.79 (17)	C22—C23—H23	119.5
C8—C9—Br1	120.08 (14)	C23—C24—C25	120.1 (2)
C10—C9—Br1	120.13 (14)	C23—C24—H24	119.9
C11—C10—C9	119.84 (18)	C25—C24—H24	119.9
C11—C10—H10	120.1	C24—C25—C26	119.8 (2)
C9—C10—H10	120.1	C24—C25—H25	120.1
C10—C11—C12	120.33 (16)	C26—C25—H25	120.1
C10—C11—C14	118.52 (17)	C21—C26—C25	119.4 (2)
C12—C11—C14	121.15 (16)	C21—C26—H26	120.3
C13—C12—C11	119.80 (16)	C25—C26—H26	120.3
C13—C12—H12	120.1		

### Hydrogen-bond geometry (Å, º)

**Table:**

*D*—H···*A*	*D*—H	H···*A*	*D*···*A*	*D*—H···*A*
C14—H14···O5	0.93	2.30	2.991 (2)	131
